# Moving object tracking in clinical scenarios: application to cardiac surgery and cerebral aneurysm clipping

**DOI:** 10.1007/s11548-019-02030-z

**Published:** 2019-07-15

**Authors:** Sarada Prasad Dakua, Julien Abinahed, Ayman Zakaria, Shidin Balakrishnan, Georges Younes, Nikhil Navkar, Abdulla Al-Ansari, Xiaojun Zhai, Faycal Bensaali, Abbes Amira

**Affiliations:** 1 Department of Surgery, Hamad Medical Corporation, Doha, Qatar; 2 Department of Electrical Engineering, Qatar University, Doha, Qatar; 3 School of Computer Science and Electronic Engineering, University of Essex, Colchester, UK; 4 Faculty of Computing, Engineering and Media De Montfort University, Leicester, UK

**Keywords:** Cerebral aneurysm, Segmentation, Object tracking, Heart surgery, Brain aneurysm clipping, Level sets

## Abstract

**Background and objectives:**

Surgical procedures such as laparoscopic and robotic surgeries are popular since they are invasive in nature and use miniaturized surgical instruments for small incisions. Tracking of the instruments (graspers, needle drivers) and field of view from the stereoscopic camera during surgery could further help the surgeons to remain focussed and reduce the probability of committing any mistakes. Tracking is usually preferred in computerized video surveillance, traffic monitoring, military surveillance system, and vehicle navigation. Despite the numerous efforts over the last few years, object tracking still remains an open research problem, mainly due to motion blur, image noise, lack of image texture, and occlusion. Most of the existing object tracking methods are time-consuming and less accurate when the input video contains high volume of information and more number of instruments.

**Methods:**

This paper presents a variational framework to track the motion of moving objects in surgery videos. The key contributions are as follows: (1) A denoising method using stochastic resonance in maximal overlap discrete wavelet transform is proposed and (2) a robust energy functional based on Bhattacharyya coefficient to match the target region in the first frame of the input sequence with the subsequent frames using a similarity metric is developed. A modified affine transformation-based registration is used to estimate the motion of the features following an active contour-based segmentation method to converge the contour resulted from the registration process.

**Results and conclusion:**

The proposed method has been implemented on publicly available databases; the results are found satisfactory. Overlap index (OI) is used to evaluate the tracking performance, and the maximum OI is found to be 76% and 88% on private data and public data sequences.

## Introduction

Looking at the steep rise in cardiac diseases, bona fide treatment including surgery is necessary to prevent its rise and avoid sudden cardiac death [1]. Similarly, cerebral aneurysm (CA) is one of the devastating cerebrovascular diseases of adult population worldwide that cause subarachnoid hemorrhage, intracerebral hematoma, and other complications leading to high mortality rate [2]. Surgery is considered as an efficient modality for the patients with cardiac complications and ruptured cerebral aneurysms. Tracking could be considered as a treatment support and planning in robotic, laparoscopic, and medical education. During robotic surgery or laparoscopic surgery, the surgeons concentrate on the surgery to avoid even slight, possible mortality and morbidity and usually get stressed. In this scenario, motion tracking of the tools and viewing the desired operating field may be considered two supportive pillars to augment the treatment and improve success rate.

### Clinical requirements in surgery

Many factors contribute to successful outcome of a surgery, specifically minimally invasive surgery (MIS). These include technical factors, such as in-depth understanding of the relevant anatomy, clear understanding of the steps involved in the procedure, well-honed surgical skills and tool manipulation, as well as anthropomorphic factors such as operating team chemistry and dynamics. To a certain degree, MIS surgeons can advance their anatomy knowledge and procedural understanding through reading and surgical videos; however, other technical skills such as tool manipulation and positioning, which are very crucial to the successful outcome of the surgery [3, 4] are more complex, nuanced and time dependent to develop due to restricted vision, limited working space, loss of visual cues and tactile feedback [5]. Quality and adequacy of surgical proficiency directly impact intra-operative and postoperative outcomes [6]. The existing “apprenticeship” model of training in surgery provides limited and time-consuming opportunities to gain the required technical competencies. In its current form, the assessment of surgical proficiency is heavily reliant on subject-matter experts/subjective assessments [3]. Thus, surgical training and planning could benefit greatly from visual support provided by instrument/motion tracking, by providing benchmarked metrics for continued objective and constructive assessment of highest standards of surgical skills, and lowering the risk of false tool trajectories and orientations [7], alignment of implants and placement of screws [8], etc.

Such augmented visual support for both surgical training and planning could be provided through object/motion tracking of the tools (such as scope, scissors, etc.) by providing objective assessment, benchmarking, and automated feedback on metrics such as path length/deviation, economy and smoothness of hand movements, depth perception, rotational orientation, changes in instrument velocity and time [9]. Zhao et al. [10] report that intra-operative tracking/detection of surgical instruments can provide important information to monitor instruments for the operational navigation in MIS, especially in the robotic minimally invasive surgeries (RMIS). Thus, based on the above, the perceived impact of tool tracking/positioning on surgical training and intra-operative guidance leads to (a) ensured patient safety via proficient tool movements and avoidance of critical tissue structures and (b) facilitation of a smooth and efficient invasive procedure [11]. This is crucial in surgery, as by continuously charting the location, movement, speed, and acceleration of the different surgical instruments in the operating field, the surgeon is continuously aware of the whereabouts of his instruments in relation to the patient’s vital organs, blood vessels, and nerves during surgery. For surgical training, it objectively helps assess surgical performance and helps differentiate between an expert and a novice surgeon, such that optimal training can then be provided to the novice to ensure the highest levels of patient care [3]. Therefore, precise positioning of the tools remains pivotal in minimally invasive surgical procedures [12] highlighting the need of object tracking via its impact on surgical training and intra-operative guidance.

Kobayashi et al. [13] applied surgical navigation techniques and tool tracking to renal artery dissection within the robot-assisted partial nephrectomy procedure and found that inefficient tool movements involving “insert,” “pull,” and “rotate” motions, as well as time to visualize and dissect the artery were significantly improved owing to improved visualization and control over the tool and anatomy. Pediatric orthopedic surgeons found an increase in accuracy and a reduction in operating time when using image-guided surgical robotic systems to overcome the inaccuracies of hand-controlled tool positioning [14]; these robots achieve this by providing information about surgical tools or implants relative to a target organ (bone). In urology, motion tracking can greatly assist in outpatient procedures such as MRI and ultrasound-guided prostate biopsy, allowing the surgeon to accurately position and invade suspicious malignant zones for a tissue sample [15]. In interventional radiology, motion tracking can help track guide-wires during endovascular interventions and radiation therapy [16]. In addition to these, applications of surgical navigation systems and tool tracking/motion analysis are being explored in many other surgical fields, including ear-nose-and-throat (ENT) surgery [7], craniomaxillofacial surgery [17], cardiothoracic surgery [18], and orthopedic surgery [19].

### Related work

The literature of motion tracking is rich; a few recent methods are included in this paper. Kim and Park [20] present a strategy that is based on edge information to assist object-based video coding, motion estimation, and motion compensation for MPEG 4 and MPEG 7 utilizing the human visual perception to provide edge information. However, the method critically depends on its ability to establish correct correspondences between points on the model edges and edge pixels in an image. Furthermore, this is a non-trivial problem especially in the presence of large inter-frame motions and cluttered environments. Subudhi et al. [21] propose a two-step method: spatio-temporal spatial segmentation and temporal segmentation that uses Markov random field (MRF) model and posteriori probability (MAP) estimation technique. Duffner and Garcia [22] present an algorithm for real-time single-object tracking, where a detector makes use of the generalized Hough transform with color and gradient descriptors; a probabilistic segmentation method is used for foreground and background color distributions. However, it is computationally expensive, especially when the number of parameters is large. It also could be erroneous because the gradient information usually leads to error when noise level is high. Li et al. [23] suggest a method within the correlation framework (CF) that models a tracker maximizing the margin between the target and surrounding background by exploiting background information effectively. They propose to train a CF by multilevel scale supervision, which aims to make CF sensitive to the target scale variation. Then the two individual modules are integrated into one framework simplifying the tracking model. However, the computational load and efficiency are still two major concerns. Mahalingam et al. [24] propose a fuzzy morphological filter and blob detection-based method for object tracking. However, the performance gets deteriorated in the presence of noise, lack of illumination, and occlusion. Zhang et al. [25] propose a correlation particle filter (CPF) that combines a correlation filter and a particle filter. However, this tracker is still unable to deal with scale variation and partial occlusion. Yang et al. [26] present a method to analyze frames extracted from videos using kernelized correlation filters (KCF) and background subtraction (BS) (KCF-BS) to plot the 3D trajectory of cabbage butterfly. The KCF-BS algorithm is used to track the butterfly in video frames and obtain coordinates of the target centroid in two videos. However, it is noticed that the target sometimes gets lost and the method is unable to re-detect or recognize the target when the target motion is fast. Du et al. [27] propose an object tracking method for satellite videos by fusing KCF tracker and a three-frame difference algorithm. Although the method reports interesting results, it takes long time to perform. Liu et al. [29] propose a correlation filter-based tracker that consists of multiple positions’ detections and alternate templates. The detection position is repositioned according to the estimated speed of target by an optical flow method, and the alternate template is stored with a template update mechanism. However, this method fails to perform if the size of each target is too small compared with the entire image, and the target and the background are very similar. Liu et al. [30] propose a method by integrating histogram of oriented gradient, RGB histogram, and motion histogram into a novel statistical model to track the target in unmanned aerial vehicle-captured videos. However, it fails to perform in occluded scenes.

Du et al. [31] present a method that is based on iterative graph seeking. Usually, the superpixel-based methods use mid-level visual cues to represent target parts where local appearance variations are exploited by superpixel representation. These methods have three sequential steps: (A) target part selection, (B) target part matching, and (C) target state estimation. (A) selects candidate target parts from the background, (B) a local appearance model associates parts between consecutive frames (target part matching, center pixel location and size of the target) is estimated based on majority voting, and (C) target state is estimated based on majority voting of matching results. This method integrates target part selection, part matching, and state estimation using a unified energy minimization framework. It incorporates structural information in local parts variations using the global constraint. Although the results are reported promising, the target part selection and target part matching when combinedly merge with the correlation filter, the estimation of the target takes long time to converge due to scale variation and partial occlusion that are bound to happen in surgery scenarios. Furthermore, when the noise level (for instance, in cardiac cine MRI data) in the input frames is high, the method would certainly struggle to perform. We intend to address these issues through our proposed method. Furthermore, if the literature above is carefully observed, noise has always been an issue in most of the methods. Therefore, in our proposed method, we first denoise the input frames. The target region on the first frame is chosen by a level set (LS) function, and then, the foreground and background models are generated. The foreground and background distributions are determined using the models in subsequent frames, and the motion of the pixels from the region of interest is estimated through a registration framework. Additionally, the selected region contour in the current frame is registered with the subsequent frame. Finally, segmentation is applied to refine the contour generated during registration and the contour is updated.

The paper is organized as follows: Section “Methodology and data” describes the denoising stage; “Target rendering” section presents the approach for target rendering (including region selection and developing models); “Registration” section defines a method for motion estimation through registration; “Segmentation” section presents the segmentation; “Results and discussion” section provides the results, while “Conclusions and future work” section concludes the paper.

**Fig. 1:**
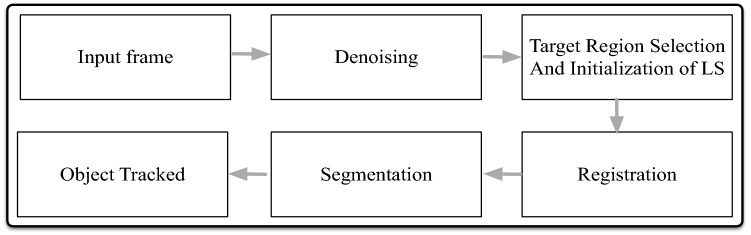
Block diagram describing the proposed method

## Methodology and data

The method is illustrated in Fig. 1. First, the input frame is denoised to minimize the negative impact of noise on subsequent steps. The target region is then selected followed by the development of background models for motion estimation through a registration framework. Finally, the rough contour generated in registration step is further refined (by a proper segmentation method) and the contour is updated on subsequent frames.

### Denoising of image sequences

Over the years, most of the methods address the noisy and cluttered medical images, mostly, by filtering that result significant degradation in image quality. One of the efficient approaches that counter noise and constructively utilize noise is stochastic resonance (SR) [33]. SR occurs if the signal-to-noise ratio (SNR) and input/output correlation have a well-marked maximum at a certain noise level. Unlike very low or high noise intensities, moderate ones allow the signal to cross the threshold giving maximum SNR at some optimum noise level. In the bistable SR model, upon addition of zero mean Gaussian noise, the pixel is transferred from weak signal state to strong signal state, which is modeled by Brownian motion of a particle (*pc*) placed in a double-well potential system. The state at which performance metrics are found optimum can be considered as the stable state providing maximum SNR. There have already been many attempts to use SR in different domains such as Fourier and spatial domains [34]; however, we have chosen the maximal overlap discrete wavelet transform (MODWT) [36] because of some of its key advantages: (1) MODWT can handle any sample size, (2) the smooth and detail coefficients of MODWT multiresolution analysis are associated with zero phase filters, (3) it is transform invariant, and (4) it produces a more asymptotically efficient wavelet variance estimator than DWT.

#### Maximal overlap discrete wavelet transform

Generally, DWT is defined by: ${\psi _{j,k}}\left( t \right) = {2^{\frac{j}{2}}}\psi \left( {{2^j}t - k} \right) \quad j, k \in {\text {Z}};\;z = \left\{ {0,1,2,\ldots } \right\}$, where $\psi$ is a real-valued function compactly supported, and $\int \nolimits _{ - \infty }^\infty {\psi \left( t \right) \hbox {d}t = 0}$. MODWT is evaluated using dilation equations: $\phi \left( t \right) = \sqrt{2} \sum \nolimits _k {{l_k}} \phi \left( {2t - k} \right) , \psi \left( t \right) = \sqrt{2} \sum \nolimits _k {{h_k}} \phi \left( {2t - k} \right)$, where $\phi \left( {2t - k} \right)$ and $\psi \left( t \right)$ are father wavelet defining low-pass filter coefficients and mother wavelet defining high-pass filter coefficients $l_k$: ${l_k} = \sqrt{2} \int \nolimits _{ - \infty } ^{\infty } \phi \left( t \right) \phi \left( 2t - k \right) \hbox {d}t, h_k = \sqrt{2} \int \nolimits _{ - \infty }^\infty {\psi \left( t \right) \psi \left( {2t - k} \right) \hbox {d}t}$.

#### Denoising by MODWT

In this methodology, 2D MODWT is applied to the $M \times N$ size image *I*. Applying SR to the approximation and detail coefficients, the stochastically enhanced (tuned) coefficient sets in MODWT domain are obtained as $W_\psi ^s \left( {l,p,q} \right) _{SR}$ and $W\left( {l_0 ,p,q} \right) _{SR}$. The SR in discrete form is defined as: $\frac{\mathrm{dx}}{\mathrm{dt}} = \left[ {ax - ex^3 } \right] + B\sin \omega t + \sqrt{D} \xi \left( t \right)$, where $\sqrt{D} \xi \left( t \right)$ and $B\sin \omega t$ represent noise and input, respectively; these are replaced by MODWT sub-band coefficients. The noise term is the factor to produce SR; maximization of SNR occurs at the double-well parameter *a*. Implementation of SR on digital images necessitates the need for solving the stochastic differential equation using Euler–Maruyama’s method [35] that gives the iterative discrete equation:

1 $$\begin{aligned} {x(n+1)} = {x(n)}+\varDelta {t}\left[ (ax(n)-ex^{3}(n))+ \hbox {Input}(n)\right] \quad \end{aligned}$$

where *a* and *e* are the bistable parameters, whereas *n* and $\varDelta {t}$ represent iteration and sampling time, respectively. *Input* denotes the sequence of input signal and noise, with the initial condition being $x(0)=0$. The final stochastic simulation is obtained after some predefined number of iterations. Given the tuned (enhanced and stabilized) set of wavelet coefficients ($X_\phi \left( {l_0 ,p,q} \right)$ and $X_\psi ^s \left( {l,p,q} \right)$), the denoised image $I_\mathrm{denoised}$ in spatial domain is obtained by inverse maximal overlap discrete wavelet transform (IMODWT) as:

$$\begin{aligned} I_\mathrm{denoised}= & {} \frac{1}{{\sqrt{MN} }}\sum \limits _p {\sum \limits _q {X_\phi \left( {l_0 ,p,q} \right) \phi _{l_0 ,p,q} \left( {i,j} \right) } } \\&+{\frac{1}{{\sqrt{MN} }}\sum \limits _{s \in \left( {H,V,D} \right) } {\sum \limits _{l = l_0 } {\sum \limits _p {\sum \limits _q {X_\psi ^s \left( {l,p,q} \right) \psi _{l_0 ,p,q}^s \left( {i,j} \right) } } } } } \\ \end{aligned}$$

The double-well parameters *a* and *e* are determined from the SNR by differentiating SR with respect to *a* and equating to zero; in this way, SNR is maximized resulting in $a = 2\sigma _0 ^2$ for maximum SNR, where $\sigma _0$ is the noise level administered to the input image. The maximum possible value of restoring force ($R = B\sin \omega t$) in terms of gradient of some bistable potential function *U*(*x*), $R = - \frac{\mathrm{dU}}{\mathrm{dx}} = - ax + ex^3 ,\frac{\mathrm{dR}}{\mathrm{dx}} = - a + 3ex^2 = 0$ resulting in $x = \sqrt{{a/{3e}}}$. *R* at this value gives maximum force as $\sqrt{\frac{{4a^3 }}{{27e}}}$ and $B\sin \omega t < \sqrt{\frac{{4a^3 }}{{27e}}}$. Maximizing the left term (keeping $B=1$), $e < \frac{{4a^3 }}{{27}}$. In order to get the parameter values, we consider $a =w \times 2\sigma _0 ^2$, and $e=z \times \sqrt{\frac{{4a^3 }}{{27}}}$; *w* and *z* are weight parameters for *a* and *e*. Initially, *w* is an experimentally chosen constant that later becomes input image standard deviation dependent, while *z* is a number less than 1 to ensure sub-threshold condition of the signal. In this way, the noise in input image is countered and maximum information from the image is achieved.

### Target rendering

Target region selection or target rendering [28, 37] is the initial step in this motion tracking. Then the features (such as intensity, color, edge, texture, etc.) are selected that can appropriately describe the target. The notations used in target rendering are: *fs*—feature space, *r*—number of features, $\mathbf fd$—foreground distribution (by the features), and $\mathbf bd$—background distribution. The region is initialized on the first frame and represented by a level set function $\phi$ because of its flexibility in choosing the contour. The distributions of foreground ($\phi \ge 0$) and background ($- th< \phi < 0$, *th* is the threshold to restrict the region of interest into small area) regions are represented by $fg\left( \phi \right)$ and $bg\left( \phi \right)$, respectively, and match with $\mathbf fd$ and $\mathbf bd$. Next, the foreground and background models are generated. Suppose the pixels ${\left\{ {{x_{f,i}}} \right\} _{i = 1,\ldots ,{n_f}}}$ and ${\left\{ {{x_{b,i}}} \right\} _{i = 1,\ldots ,{n_b}}}$ fall in foreground and background regions; the function $z:{\mathfrak {R}^2} \rightarrow \left\{ {1,\ldots ,r} \right\}$ can be used to map the pixels ($x_i$) into the bin $b(x_i)$ in feature space. The probability of the feature space in the models is: $f{d_{fs}} = \frac{1}{{{n_f}}}\sum \nolimits _{i = 1}^{{n_f}} {\delta \left[ {\left( {{x_{i,f}}} \right) - fs} \right] }$ and $b{d_{fs}} = \frac{1}{{{n_b}}}\sum \nolimits _{i = 1}^{{n_b}} {\delta \left[ {\left( {{x_{i,f}}} \right) - fs} \right] }$, where $\delta$ is the Kronecker delta function and $n_f$ and $n_b$ are the number of pixels in foreground and background, respectively. The foreground and background distributions in the current frame candidate region ($- th< \phi < 0$) are obtained as:

2 $$\begin{aligned} fg\left( \phi \right)= & {} \frac{1}{{{F_f}}}\sum \limits _{i = 1}^n {H\left( {\phi \left( {{x_i}} \right) } \right) \delta \left[ {b\left( {{x_i}} \right) - fs} \right] } \;\hbox {and}\;bg\left( \phi \right) \nonumber \\= & {} \frac{1}{{{F_b}}}\sum \limits _{i = 1}^n {\left( {1 - H\left( {\phi \left( {{x_i}} \right) } \right) } \right) \delta \left[ {b\left( {{x_i}} \right) - fs} \right] } \end{aligned}$$

*H*(.) is the Heaviside function to select foreground region; ${F_f}$ and ${F_b}$ are the normalization factors.

### Registration

Registration of the target in the first frame with the next subsequent frame is performed to estimate the affine deformation of the target. We determine the foreground and background distributions in the frames and match them with respective foreground and background models. We use Bhattacharyya metric [38] because it is computationally fast and is already being used in face recognition for years. Additionally, it has straightforward geometric interpretation. Since it is the cosine angle between $\mathbf fd$ and $fg(\phi )$ or between $\mathbf bd$ and $bg(\phi )$, higher value of the coefficient indicates better matching between candidate and target models. Thus, our similarity distance measure:

3 $$\begin{aligned} \begin{array}{*{20}c} \begin{array}{l} E{n_1}\left( \phi \right) =\sum \limits _{fs=1}^r {\left( {\sqrt{f{g_{fs}}\left( \phi \right) f{d_{fs}}} + \gamma \sqrt{b{g_{fs}}\left( \phi \right) b{d_{fs}}} } \right) }\qquad \end{array} \end{array} \end{aligned}$$

where $\gamma$ is the weight to balance the contribution from both foreground and background in the matching.

For deformation estimation, we have proposed a simple and efficient framework as follows. Suppose in the current frame, $\phi _0$ is the target initial position and the contour is obtained by $\phi =0$. The probabilities $fg\left( {{\phi _0}} \right) = {\left\{ {f{g_{fs}}\left( {{\phi _0}} \right) } \right\} _{fs = 1,\ldots ,r}}$ and $bg\left( {{\phi _0}} \right) = {\left\{ {b{g_{fs}}\left( {{\phi _0}} \right) } \right\} _{fs = 1,\ldots ,r}}$ are computed. Applying Taylor’s expansion:

4 $$\begin{aligned} E{n_1}\left( \phi \right)= & {} \frac{1}{2}\left( {\sum \limits _{fs = 1}^r {\sqrt{f{g_{fs}}\left( {{\phi _0}} \right) f{d_{fs}}} + \sum \limits _{fs = 1}^r {f{g_{fs}}\left( \phi \right) } \sqrt{\frac{{f{d_{fs}}}}{{f{g_{fs}}\left( {{\phi _0}} \right) }}} } } \right) \nonumber \\&\quad + \frac{1}{2}\gamma \left( {\sum \limits _{fs = 1}^r {\sqrt{b{g_{fs}}\left( {{\phi _0}} \right) b{d_{fs}}} + \sum \limits _{fs = 1}^r {b{g_{fs}}\left( \phi \right) } \sqrt{\frac{{b{d_{fs}}}}{{b{g_{fs}}\left( {{\phi _0}} \right) }}} } } \right) \nonumber \\ \end{aligned}$$

By putting Eq. (2) in (4), we get:

5 $$\begin{aligned} E{n_1}\left( \phi \right)= & {} \frac{1}{2}\left( {\sum \limits _{fs = 1}^r {\sqrt{f{g_{fs}}\left( {{\phi _0}} \right) f{d_{fs}}} + \frac{1}{{{F_f}}}\sum \limits _{fs = 1}^n {{h_{f,i}}H\left( {\phi \left( {{x_i}} \right) } \right) } } } \right) \nonumber \\&+ \frac{1}{2}\gamma \left( {\sum \limits _{fs = 1}^r {\sqrt{b{g_{fs}}\left( {{\phi _0}} \right) b{d_{fs}}} + \frac{1}{{{F_b}}}\sum \limits _{fs = 1}^n {{h_{b,i}}\left( {1 - H\left( {\phi \left( {{x_i}} \right) } \right) } \right) } } } \right) \nonumber \\ \end{aligned}$$

where the weights that play a pivotal role in detecting the new centroid of the target are: $${h_{f,i}} = \sum \nolimits _{fs = 1}^r$$$${\sqrt{\frac{{f{d_{fs}}}}{{f{g_{fs}}\left( {{\phi _0}} \right) }}} \delta \left[ {z\left( {{x_i}} \right) - fs} \right] } \;and\;{h_{f,i}} = \sum \nolimits _{fs = 1}^r {\sqrt{\frac{{b{d_{fs}}}}{{b{g_{fs}}\left( {{\phi _0}} \right) }}} \delta \left[ {z\left( {{x_i}} \right) - fs} \right] } $$. Higher value of Bhattacharyya coefficient can be obtained by maximizing (5) that is a function of location *x* and contour.

Furthermore, we consider the foreground and background intensity as additional feature. Suppose the first frame, $u_0(x,y)$, consists of two concentric regions ($u_0^i$, $u_0^o$) meaning the input image contains more than one intensity label. This is certainly challenging in determining a smooth contour initialization and deformation because of varying intensities. Therefore, we integrate both local and global image information in the energy term in order to make it perform as a perfect step detector with respect to the initialization of contour. The energy term is defined as:

6 $$\begin{aligned} {En_2} = {\lambda _1}{E^G} + {\lambda _2}{E^L} + {E^R} \end{aligned}$$

where $\lambda _1$ and $\lambda _2$ are fixed constants; $E^G$, $E^L$, and $E^R$ are the global term, local term, and regularized term, respectively (containing respective image information). $E^R$ controls the boundary smoothness. The local term is defined as,

7 $$\begin{aligned}&E^L= \int \limits _{\phi < 0} {\frac{{{{\left( {{g_k}{u_0}\left( {x,y} \right) - {u_0}\left( {x,y} \right) - {d_1}\left( {x,y} \right) } \right) }^2}}}{{{d_1}{{\left( {x,y} \right) }^2}}}\hbox {d}x\hbox {d}y} \nonumber \\&\qquad \quad +\int \limits _{\phi > 0} {\frac{{{{\left( {{g_k}{u_0}\left( {x,y} \right) - {u_0}\left( {x,y} \right) - {d_2}\left( {x,y} \right) } \right) }^2}}}{{{d_2}{{\left( {x,y} \right) }^2}}}\hbox {d}x\hbox {d}y} \nonumber \\ \end{aligned}$$

where $g_k$ is an averaging filter with $k \times k$ size, $d_1$ and $d_2$ are intensity averages of the difference image ${g_k}{u_0}\left( {x,y} \right) - {u_0}\left( {x,y} \right)$ inside and outside the variable curve *C*, respectively. The global term:

8 $$\begin{aligned} \begin{array}{*{20}{c}} {E^G =\displaystyle \int \limits _{\phi <0} {\frac{{{{\left( {{u_0}\left( {x,y} \right) - {c_1}\left( {x,y} \right) } \right) }^2}}}{{{c_1}{{\left( {x,y} \right) }^2}}}\hbox {d}x\hbox {d}y} } { +\int \limits _{\phi >0} {\frac{{{{\left( {{u_0}\left( {x,y} \right) - {c_2}\left( {x,y} \right) } \right) }^2}}}{{{c_2}{{\left( {x,y} \right) }^2}}}\hbox {d}x\hbox {d}y} } \end{array} \end{aligned}$$

where the constants $c_1$, $c_2$ represent the average intensity of $u_0(x,y)$ inside *C* and outside *C*, respectively. $c_1$ and $c_2$ are approximated by a weighted average of image intensity ${u_0}\left( {p,q} \right)$, where (*p*, *q*) is the neighborhood of (*x*, *y*). It means ${c_1}\left( {x,y} \right)$ and ${c_2}\left( {x,y} \right)$ are spatially varying; we formulate ${c_1}\left( {x,y} \right)$ and ${c_2}\left( {x,y} \right)$ as, ${c_1}\left( {x,y} \right) = {\frac{{\int \limits _\varOmega {{g_k}\left( {\left( {x,y} \right) - \left( {p,q} \right) } \right) {u_0}\left( {p,q} \right) H\left( {\phi \left( {p,q} \right) } \right) dpdq} }}{{{g_k}\left( {\left( {x,y} \right) - \left( {p,q} \right) } \right) H\left( {\phi \left( {p,q} \right) } \right) dpdq}}}$ and ${c_2}\left( {x,y} \right) = {\frac{{\int \limits _\varOmega {{g_k}\left( {\left( {x,y} \right) - \left( {p,q} \right) } \right) {u_0}\left( {p,q} \right) \left( {1 - H\left( {\phi \left( {p,q} \right) } \right) } \right) dpdq} }}{{{g_k}\left( {\left( {x,y} \right) - \left( {p,q} \right) } \right) \left( {1 - H\left( {\phi \left( {p,q} \right) } \right) } \right) dpdq}}}$. We use the conventional regularizing term $E_R$ that includes a penalty term on the total length of the edge contour for a given segmentation. Also it contains another penalty term on the total area of the foreground region found by the segmentation. The energy term therefore becomes:

9 $$\begin{aligned} {En_2(\phi )}= & {} \mu \int \limits _\varOmega {\delta \left( \phi \right) } + v\int \limits _\varOmega {H\left( {\phi \left( {x,y} \right) } \right) \hbox {d}x\hbox {d}y + \left| {\nabla \phi } \right| } \hbox {d}x\hbox {d}y \nonumber \\&+ {{\lambda _1}\int \limits _\varOmega {\frac{{{{\left( {{u_0}\left( {x,y} \right) - {c_1}\left( {x,y} \right) } \right) }^2}H\left( {\phi \left( {x,y} \right) } \right) }}{{{c_1}{{\left( {x,y} \right) }^2}}}\hbox {d}x\hbox {d}y} }\nonumber \\&+{{\lambda _1}\int \limits _\varOmega {\frac{{{{\left( {{u_0}\left( {x,y} \right) - {c_1}\left( {x,y} \right) } \right) }^2}\left( {1 - H\left( {\phi \left( {x,y} \right) } \right) } \right) }}{{{c_2}{{\left( {x,y} \right) }^2}}}\hbox {d}x\hbox {d}y} }\nonumber \\&+ {\lambda _2}\frac{{{{\left( {{g_k}{u_0}\left( {x,y} \right) - {d_1}\left( {x,y} \right) } \right) }^2}H\left( {\phi \left( {x,y} \right) } \right) }}{{{d_1}{{\left( {x,y} \right) }^2}}}\hbox {d}x\hbox {d}y \nonumber \\&+ {\lambda _2}\frac{{{{\left( {{g_k}{u_0}\left( {x,y} \right) - {d_2}\left( {x,y} \right) } \right) }^2}\left( {1 - H\left( {\phi \left( {x,y} \right) } \right) } \right) }}{{{d_2}{{\left( {x,y} \right) }^2}}}\hbox {d}x\hbox {d}y \nonumber \\ \end{aligned}$$

This Eq. (9) has to be maximized to obtain higher Bhattacharyya coefficient. The similarity distance measure now becomes:

10 $$\begin{aligned} \begin{array}{*{20}{c}} En\left( \phi \right) = E{n_1}\left( \phi \right) + E{n_2}\left( \phi \right) \end{array} \end{aligned}$$

We model the motion of the target as affine transformation by introducing a wrap in (10):

11 $$\begin{aligned} \begin{array}{*{20}{c}} x = h\left( {x,\varDelta T} \right) = \left( {\begin{array}{*{20}{c}} {1 + f{g_1}}&{}\quad {f{g_3}}&{}\quad {f{g_5}}\\ {f{g_2}}&{}\quad {1 + f{g_4}}&{}\quad {f{g_6}} \end{array}} \right) \left( {\begin{array}{*{20}{c}} x\\ y\\ 1 \end{array}} \right) \end{array} \nonumber \\ \end{aligned}$$

The column vector characterizes the change in poses. Substituting (11) in (10) and omitting the terms that are not a function of $\varDelta T$-incremental warp (represented $\phi$), we obtain:

12 $$\begin{aligned} En\left( \phi \right)= & {} \frac{1}{{2{F_f}}}\sum \limits _{i = 1}^n H\left( {\phi \left( {h\left( {x,\varDelta T} \right) } \right) } \right) {w_{f,i}} \nonumber \\&+ \frac{1}{{2{F_b}}}\gamma \sum \limits _{i = 1}^n {\left( {1 - H\left( {\phi \left( {h\left( {x,\varDelta T} \right) } \right) } \right) } \right) } {w_{b,i}} \end{aligned}$$

$\varDelta T$ tends to 0, and the estimation gets converged. In this way, the registration step iteratively estimates the shape change until it gets converged.

**Fig. 2:**
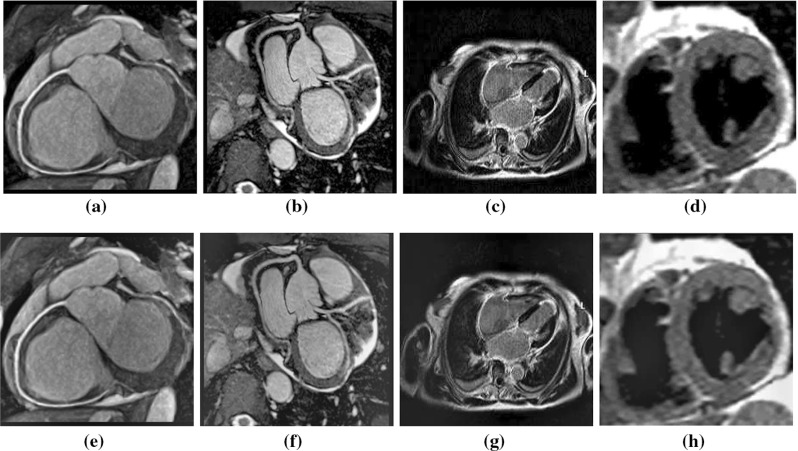
**a**–**d** Input frames in a video sequence to be denoised. **e**,**f** Results of denoising

### Segmentation

Since the tracker in the registration stage is still not able to extract the target contour properly, the registration result needs to be refined through segmentation. In order to do this, we optimize $\phi$ in Eq. (10) because the equation is a function of $\phi$; in other words, $\frac{{\partial En\left( {\phi \left( {{x_i}} \right) } \right) }}{{\partial \phi \left( {{x_i}} \right) }} = 0$. This is solved by well-known steepest-ascent method: $\frac{{\partial En\left( {\phi \left( {{x_i}} \right) ,t} \right) }}{{\partial t}} = \frac{{\partial En\left( {\phi \left( {{x_i}} \right) } \right) }}{{\partial \phi \left( {{x_i}} \right) }}$. We obtain:

13 $$\begin{aligned} \frac{{\delta \phi \left( {x,y,t} \right) }}{{\delta t}}= & {} {\delta _ \in }\left( \phi \right) \left[ \mu \nabla .\left( {\frac{{\nabla \phi }}{{\left| {\nabla \phi } \right| }}} \right) - v \nonumber \right. \\&\left. \quad + \lambda _1 \right. \left. {\left( {\frac{{{{\left( {{u_0}\left( {x,y} \right) - {c_2}\left( {x,y} \right) } \right) }^2}}}{{{c_2}{{\left( {x,y} \right) }^2}}} - \frac{{{{\left( {{u_0}\left( {x,y} \right) - {c_1}\left( {x,y} \right) } \right) }^2}}}{{{c_1}{{\left( {x,y} \right) }^2}}}} \right) } \right] \nonumber \\&\quad +{{\lambda _2}\left( {\frac{{{{\left( {{g_k}{u_0}\left( {x,y} \right) - {d_2}\left( {x,y} \right) } \right) }^2}}}{{{d_2}{{\left( {x,y} \right) }^2}}} - \frac{{{{\left( {{g_k}{u_0}\left( {x,y} \right) - {d_1}\left( {x,y} \right) } \right) }^2}}}{{{d_1}{{\left( {x,y} \right) }^2}}}} \right) }\nonumber \\&\quad + \frac{1}{2}\varDelta t{\delta _ \in }\left( \phi \right) \left( {\frac{1}{{{F_f}}}{h_{f,i}} - \gamma \frac{1}{{{F_b}}}{h_{b,i}}} \right) \end{aligned}$$

14 $$\begin{aligned}&\frac{{\partial _\varepsilon \left( \phi \right) }}{{\left| {\nabla \phi } \right| }}\frac{{\partial \phi }}{{\partial \overrightarrow{n} }} = 0\,\,on\,\,\partial \varOmega \end{aligned}$$

where *H* and $\delta _\in$ represent the Heaviside function and Dirac measure, respectively; $\frac{{\partial \phi }}{{\partial \overrightarrow{n} }}$ and ${\overrightarrow{n} }$ denote the normal derivative of $\phi$ at the boundary and the exterior normal to the boundary, respectively. Finally, the target is updated on subsequent frames.

### Data

The datasets used in this work are obtained from private sources such as Hamad Medical Corporation (30 data sequences) and public sources such as Sunnybrook [32] (45 data sequences) and VOT 2015 [40] (60 data sequences). The Sunnybrook Cardiac Data (SCD) consist of cine MRI data from a mixed set of patients and pathologies: healthy, hypertrophy, heart failure with infarction, and heart failure without infarction. Subset of this data set was first used in the automated myocardium segmentation challenge from short-axis MRI. The VOT 2015 sequences are chosen from a large pool of sequences including ALOV, OTB, non-tracking, Computer Vision Online, Professor Bob Fisher’s Image Database, Videezy, Center for Research in Computer Vision, University of Central Florida, USA, NYU Center for Genomics and Systems Biology, Data Wrangling, Open Access Directory and Learning and Recognition in Vision Group, INRIA. The initial pool of sequences is created by combining the sequences from all the sources. After removal of duplicate sequences, grayscale sequences and sequences that contained objects with area smaller than 400 pixels, the final sequences are obtained; more details can be obtained from the Web site [45].

**Fig. 3:**
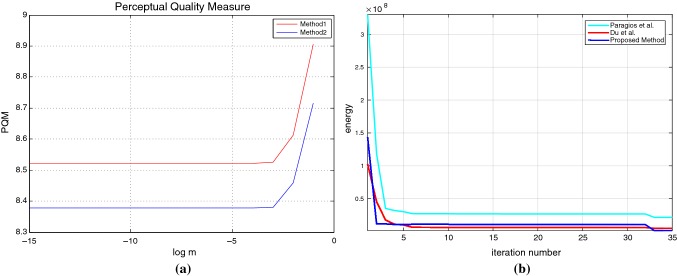
Perceptual quality measures by Fourier (Method 2) and MODWT (Method 1); *m* in x-axis denotes the mass of the particle that moves under stochastic condition. **b** Energy convergence comparison of three methods

**Fig. 4:**
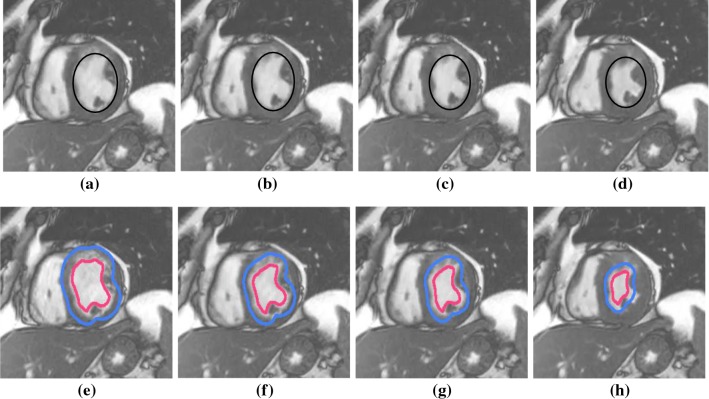
**a**–**d** Ground truth frames. **e**–**h** Tracking of left ventricle in low-contrast cine magnetic resonance imaging (low-contrast CMRI) during cardiac surgery

## Results and discussion

### Results

The proposed method is implemented on both private and public databases as described earlier. The qualitative results of denoising are provided in Fig. 2. We have quantitatively compared the proposed denoising method with that of Fourier because of its huge popularity [34]. The perceptual quality measurement (PQM) [41] is provided in Fig. 3, which shows greater value in case of MODWT suggesting higher efficacy of MODWT; in this figure, *m* denotes the mass of the particle that moves under stochastic condition. For denoising of the input images, the initial values of $\varDelta {t}$ and *z* are taken as 0.007 and 0.000027, respectively. To determine the quality of the denoised image, we have calculated distribution separation measure that estimates the degree of image quality. The DSM is defined as [34]: $\hbox {DSM} = \left| {\mu _T^E - \mu _B^E} \right| - \left| {\mu _T^O - \mu _B^O} \right|$, where $\mu _T^E$ and $\mu _T^O$ are the mean of the selected target regions of the denoised and original images, respectively; $\mu _B^E$ and $\mu _B^O$ are the mean of the selected background region of the denoised and original image, respectively. The higher the value of DSM, the better is the quality. It is observed that the value of DSM is maximum at iteration 200 and then it starts decreasing; therefore, this iteration is considered as the optimal.

**Fig. 5:**
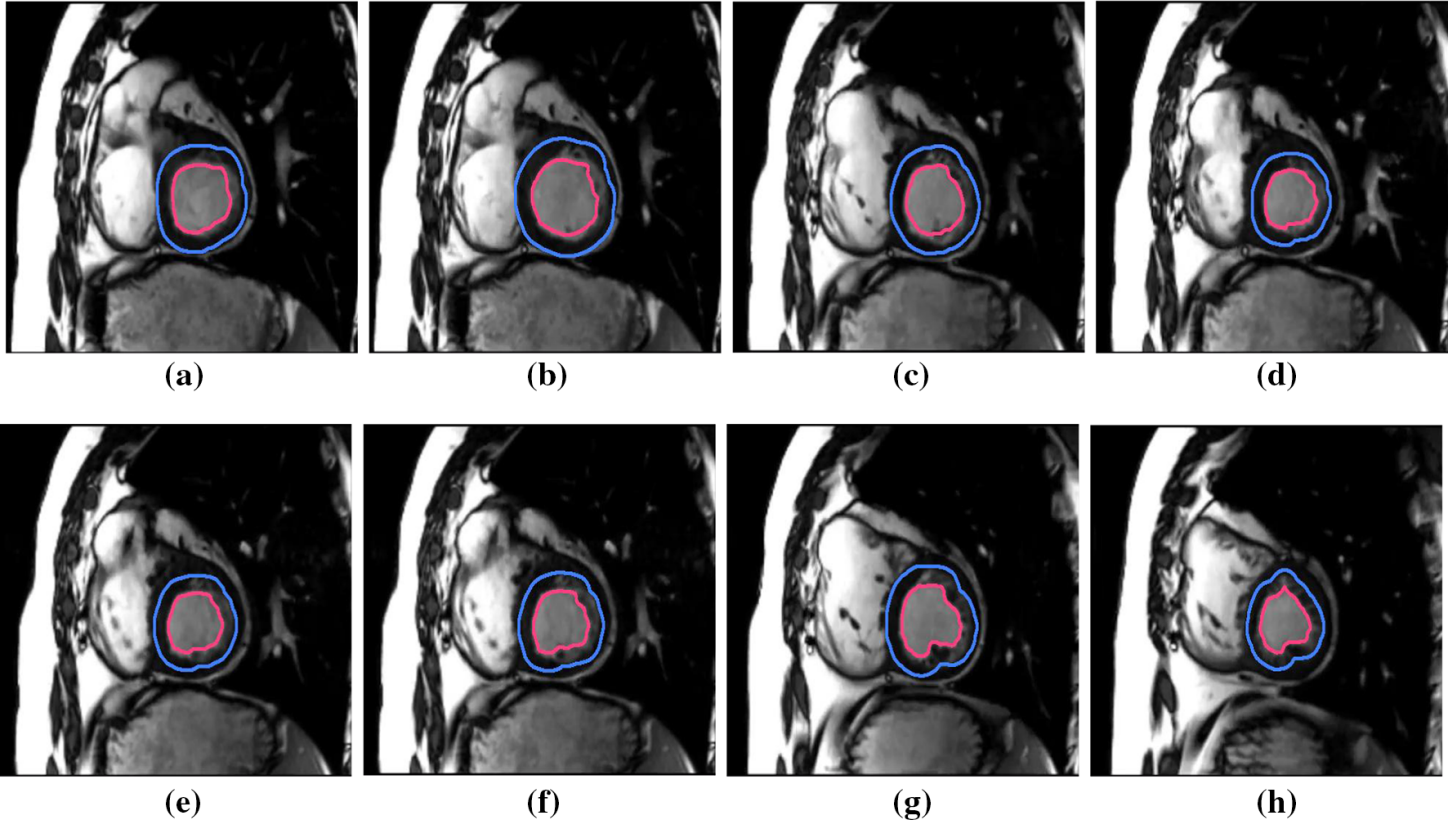
Tracking of left ventricle in high-contrast cine magnetic resonance imaging (high-contrast CMRI) during cardiac surgery

**Fig. 6:**
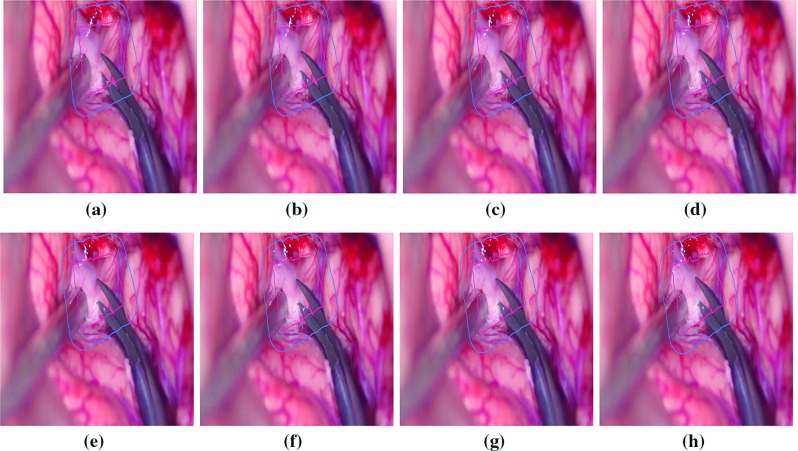
Tracking of the operating field with multiple objects during cerebral aneurysm clipping

**Fig. 7:**
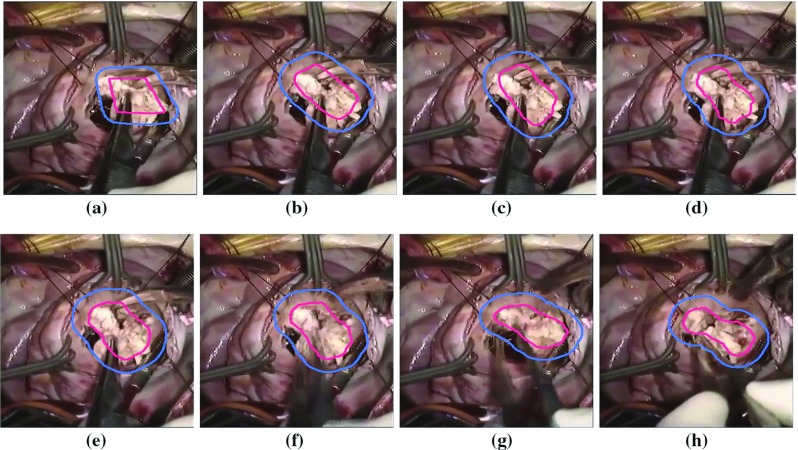
Tracking of the operating field with multiple objects during cardiac surgery

**Fig. 8:**
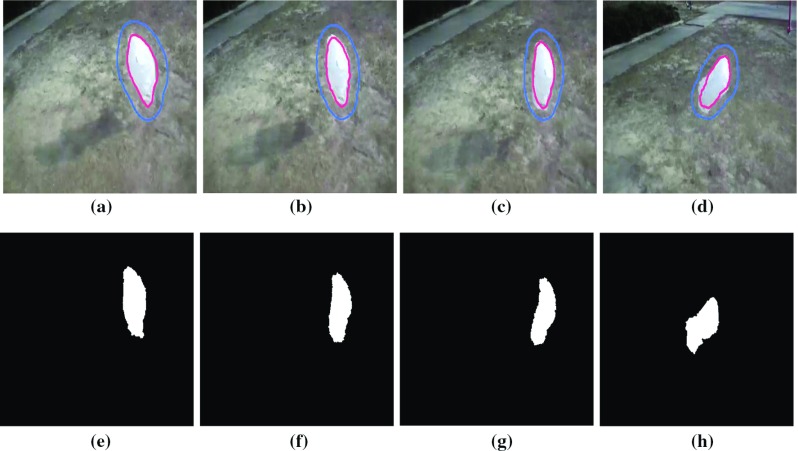
**a**–**d** Tracked frames in a video sequence (VOT 2015). **e**–**f** Corresponding ground truth sequences

These denoised frames are further used in the subsequent steps in the proposed method. As mentioned earlier, we have included the image sequences of cardiac surgery and clipping for ruptured cerebral aneurysms in this work. We have also tested our method on cardiac cine MRI datasets, high contrast and low contrast levels, to highlight the performing capability of the method in varying intensities. The performance results on these datasets are provided in Figs. 4 and 5. We have chosen different scenarios for cerebral aneurysm surgical procedure (clipping): One is to track the scissors’ or clippers’ movement and the other one is to focus on the operating field during surgery, where multiple tools are used by the surgeons. It is important to track the motion of the scissors in order to minimize the damage caused by their movement. Besides the tools’ tracking, capturing or tracking the operating field is also important; it helps the surgeon in concentrating on the tools used during the surgery and the impacted tissues of interest. The results are given in Figs. 6 and 7. We have also tested the proposed method on VOT 2015 datasets and found some satisfactory results as can be observed in Fig. 8. We have included this particular dataset in this paper to emphasize on the fact that the foreground is not very significantly different than the background like it happens in medical data sequences. Usually, the medical data are blurry (either reddish or grayish) and lack contrast as can be observed from the figures. In this scenario, only a contour surrounding the tools could easily be ignored; therefore, just for user’s (surgeon) convenience, we have added the blue line surrounding the red line in the tracking results. While calculating the accuracy, red line is only taken into consideration. In order to determine the segmentation accuracy, we have used Dice coefficient (DC), which may be defined as [44]: $DC = 2 \times \frac{{\left| {X \cap Y} \right| }}{{\left| X \right| + \left| Y \right| }}$, where *X* and *Y* are two point sets. The average segmentation accuracy on 3-T machine is 94%, whereas in case of 7 T, it is found to be 96%. The proposed method has performed as expected, which can be verified from the results provided in “Results” section. We have optimized the algorithm and code; average time taken to perform tracking and average number of frames are less than 25–30 s and 24 frames per second, respectively. We have also compared the performance of the proposed method with other similar methods ([31, 42]); the proposed method converges faster than the other methods 3(b). We have also calculated overlap index (OI) [43] to determine the overlap between the resulting target contour and the actual boundary. We have found it highest in case of the proposed method against others as can be observed from Table 1.

**Table 1:** Overlap index comparison of different methods on hospital and VOT 2015 datasets

Method	Hospital and SCD datasets			
	FOV-CA (%)	Scissors-CA (%)	Low-contrast CMRI (%)	High-contrast CMRI (%)
Paragois et al. [42]	64	65	64	65
Du et al. [31]	67	69	68	72
Proposed method	**72**	**74**	**73**	**76**

Method	VOT 2015 Dataset			
	Rabit (%)	Shaking (%)	Racing (%)	Octopus(%)
Paragois et al. [42]	69	70	68	71
Du et al. [31]	72	75	70	76
Proposed method	**83**	**85**	**82**	**88**

The results of the proposed methods are shown in bold

### Discussion

The values of bistable system parameters play a crucial role in the process of denoising using SR. The expression for SR on any data set contains additive terms of multiples of *w* and subtractive term of multiples of *z*. This is observed that the images that have low contrast and low dynamic range require larger values of *w*, while those that have relatively more contrast and cover an appreciable gray level range require smaller values of *w* for proper denoising. Values of $\varDelta {t}$ have been studied to be similar to that of *w*. This is also perceived that *w* is inversely proportional to overall variance signifying the contrast of input image. Optimization process leads us to the optimum value of *w*; the value of *z* should be less than 1 so that condition $e < \sqrt{\frac{{4a^3 }}{{27}}}$ holds assuring that the system is bistable and signal is sub-threshold so that SR can be applicable. We prefer a very small value of this factor to remain well within the allowable range of *e*. Finally, we have noticed that the varying segmentation accuracy depends on the quality of the input data sequence. The MRI data obtained from 7-T machine give better accuracy than 3-T MRI machine.

## Conclusions and future work

A variational framework has been presented to track the motion of moving objects and field of view in surgery sequences. We have presented a method that has used SR to denoise the input frames and a combined registration–segmentation framework to conduct motion tracking. We have introduced a robust similarity metric and an efficient energy functional in this framework. Despite the fact that the input data contain varying illumination, motion blur, lack of image texture, occlusion, and fast object movements, the performance of the proposed method is found quite satisfactory. In future, we intend to extensively evaluate the method quantitatively so that it can be well tested before trying in clinical practice.
